# Supplementary material for: Conditional chemoconnectomics (cCCTomics) as a strategy for efficient and conditional targeting of chemical transmission
Source: eLife. 2024 Apr 30;12:RP91927. doi: 10.7554/eLife.91927 (PMC11060718; doi:10.7554/eLife.91927)
Supplement: Figure 3—source data 3. [file elife-91927-fig3-data3.zip › Figure 3 source data3 part 1.zip!/Figure 3-Source data 3-part1/1063f3-1-CRISPResso_Report_kboyr13q.zip!/CRISPResso_on_1063f3_1/1c.Alignment_barplot.pdf]

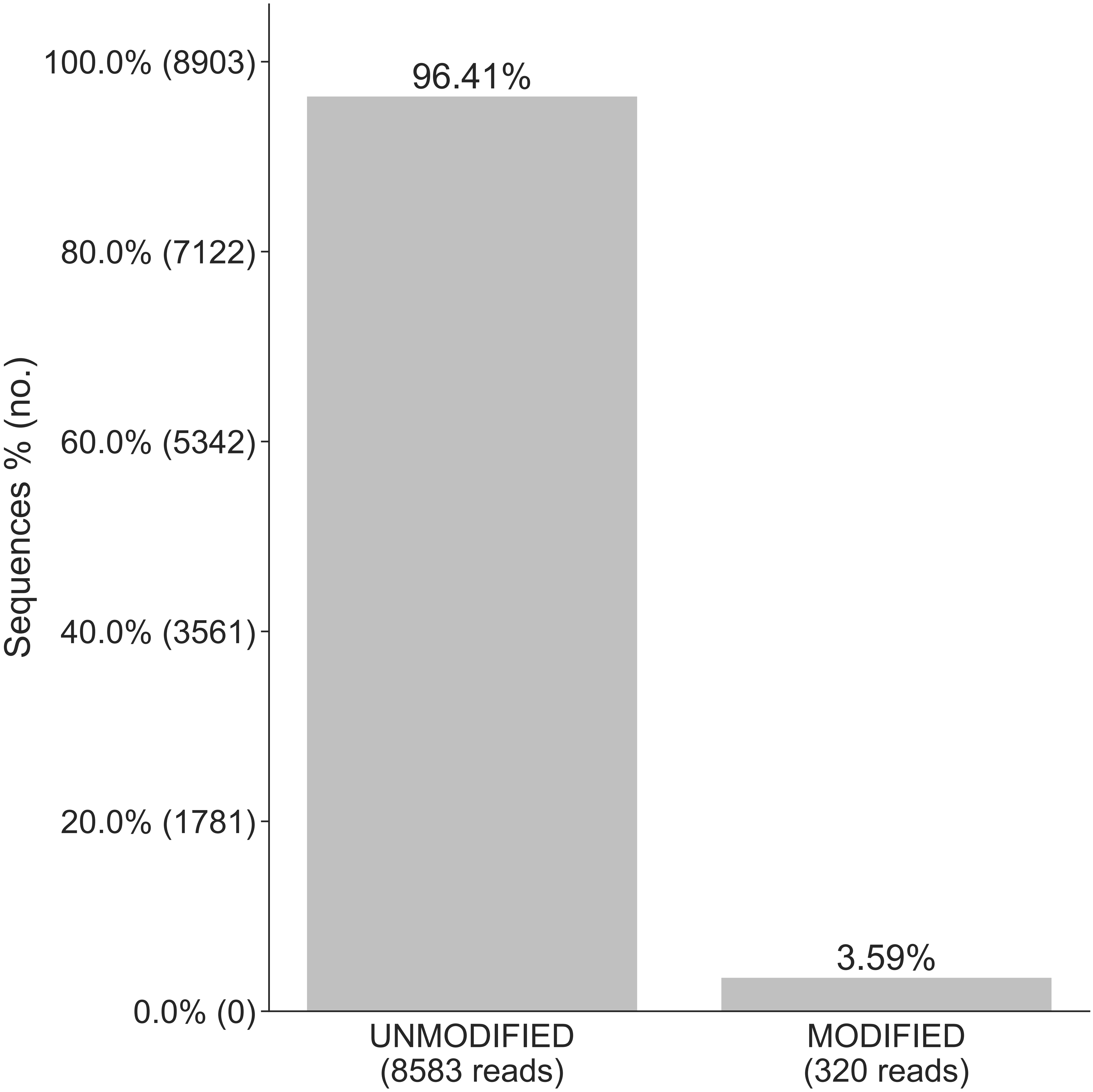
Sequences % (no.)
100.0% (8903)
80.0% (7122)
60.0% (5342)
40.0% (3561)
20.0% (1781)
0.0% (0)
96.41%
3.59%
UNMODIFIED
(8583 reads)
MODIFIED
(320 reads)
